# Supplementary material for: Song Familiarity Relies on Evidence Accumulation
Source: Psychophysiology. 2026 Jul 31;63(8):e70370. doi: 10.1111/psyp.70370 (PMC13428188; doi:10.1111/psyp.70370)
Supplement: Supplementary file 1 — Figure S1: Pooled participant response‐times (RTs) for familiar unidentified songs and familiar identified songs relative to start of song (a) and previous note (b). Dashed line indicates the mean RT for each condition. Table S1: Mean response times for unidentified and identified songs. Figure S2: Stimulus‐locked event‐related potentials (no overlap correction). Figure S3: Response‐locked event‐related potential (no overlap correction). Shaded area and scalp topography mirror the same analysis window as the main analysis. Figure S4: Design matrices for one participant's main analysis (a) and parametric analysis (b). Only the first 20,000 EEG samples are shown. Figure S5: Participant rERP scores from the main manuscript. Error bars represent 95% confidence intervals. [file PSYP-63-e70370-s001.pdf]

Supplement to *Song Familiarity Relies on Evidence Accumulation*

Stimulus/Response Overlap

Response times for 18 participants were pooled to estimate the amount of stimulus/response overlap.

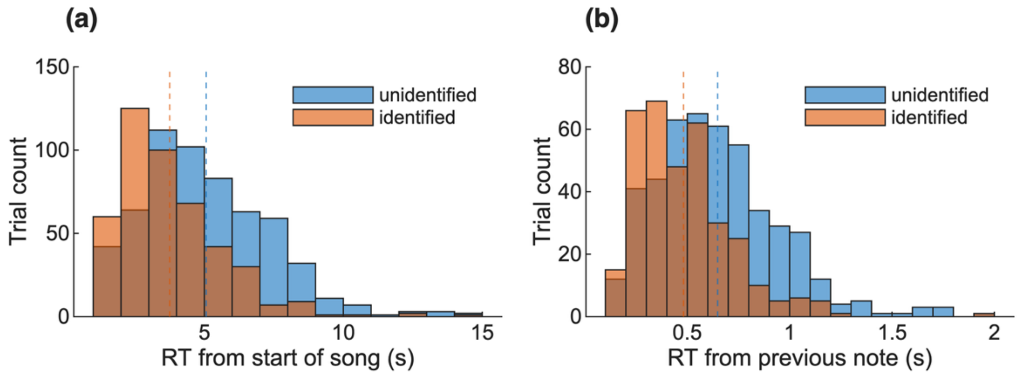

Supplementary Figure 1. Pooled participant response-times (RTs) for familiar unidentified songs and familiar identified songs relative to start of song (a) and previous note (b). Dashed line indicates the mean RT for each condition.

We also compared the mean response time for familiar unidentified songs and familiar identified songs. Response time was defined in two ways: from the start of song playback and from the onset of the note just prior to a button press.

Supplementary Table 1

Mean response times for unidentified and identified songs.

	Unidentified		Identified	
	Mean (s)	95% CI	Mean (s)	95% CI
Song start	4.99	[4.52, 5.45]	3.67	[3.30, 4.04]
Previous note	0.67	[0.61, 0.73]	0.50	[0.45, 0.55]

Familiarity decisions were significantly faster for songs that could be identified, regardless of how we defined response: $p < .001$, $t(17) = 7.83$, Cohen's $d = 1.85$ (song onset), $p < .001$, $t(17) = 7.07$, Cohen's $d = 1.67$ (previous note onset).

Traditional ERP Analysis

To visualize the effect of deconvolution, we also conducted a traditional ERP analysis. Preprocessed EEG from the main analysis was epoched around each event of interest (unfamiliar and familiar notes: -200 ms to 800 ms; responses: -1200 to 100 ms). Epochs were baseline corrected (-200 to 0 for notes, -1200 to -1000 for responses) and checked for artifacts using the following criteria: an absolute value exceeding 100 μV, a difference (maximum minus minimum) exceeding 100 μV, a sample-to-sample change exceeding 40μV, and low activity (absolute value below 0.1 μV). After removing artifactual epochs, ERPs time-locked to familiar notes, unfamiliar notes, and button presses were computed using a traditional averaging procedure (see Supplementary Figures 2 and 3 below).

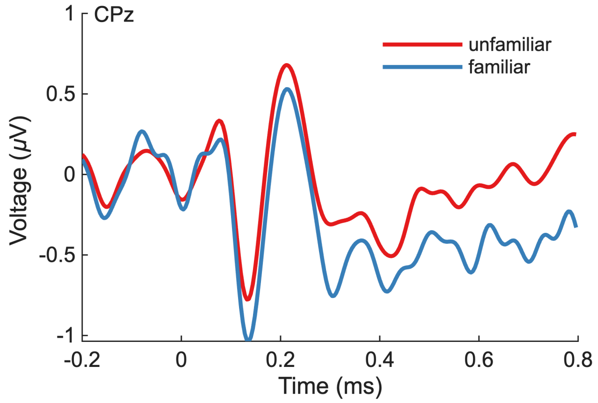

Supplementary Figure 2. Stimulus-locked event-related potentials (no overlap correction).

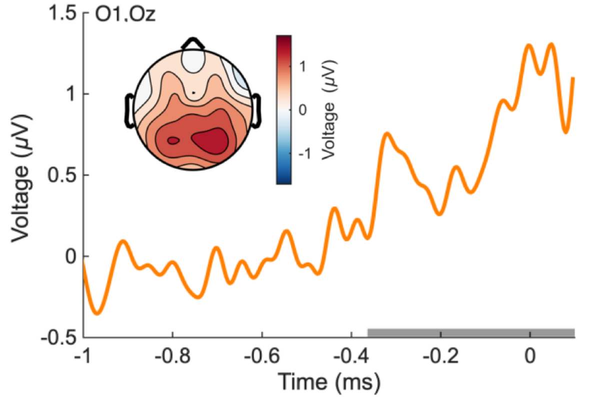

Supplementary Figure 3. Response-locked event-related potential (no overlap correction). Shaded area and scalp topography mirror the same analysis window as the main analysis.

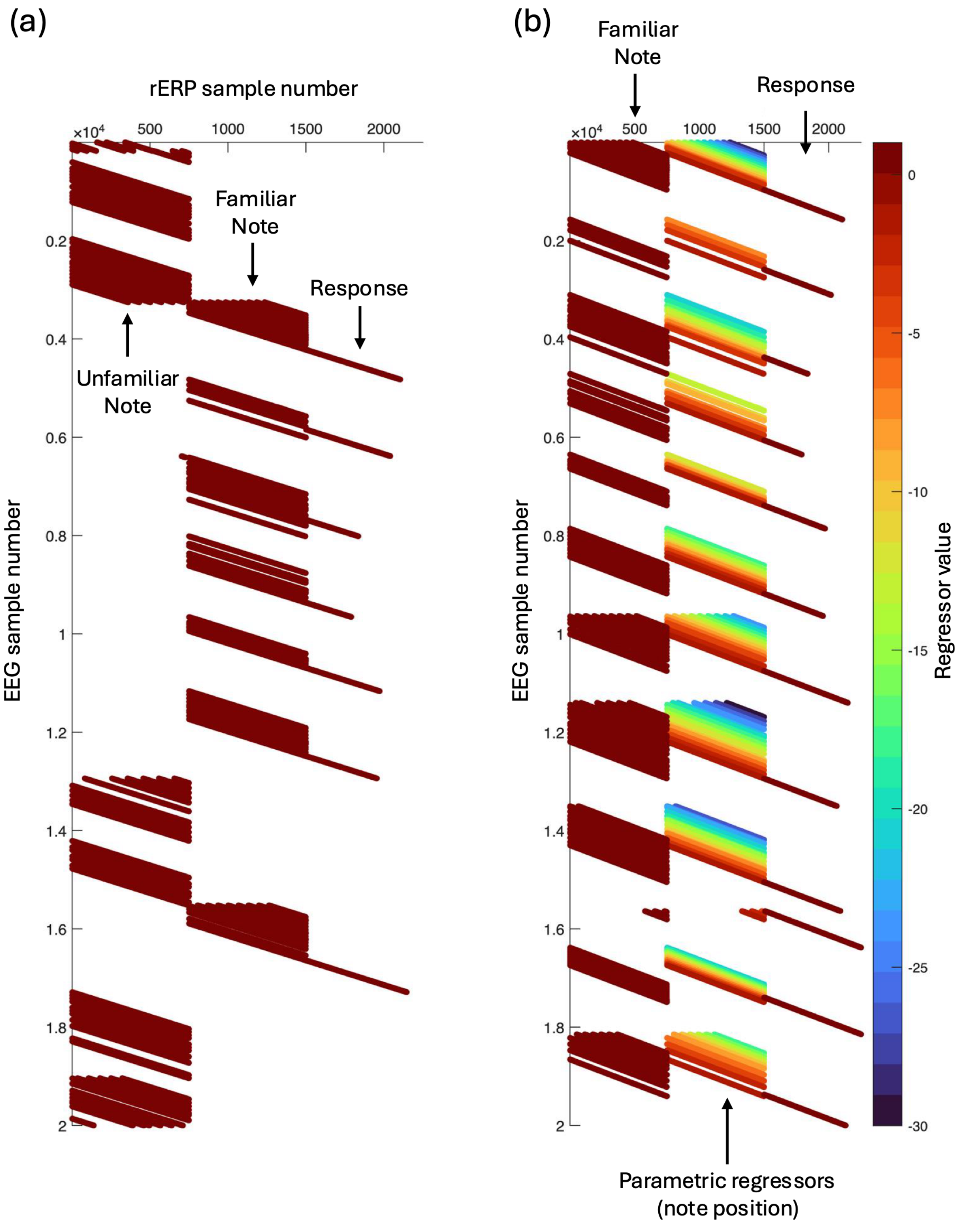

Supplementary Figure 4. Design matrices for one participant's main analysis (a) and parametric analysis (b). Only the first 20,000 EEG samples are shown.

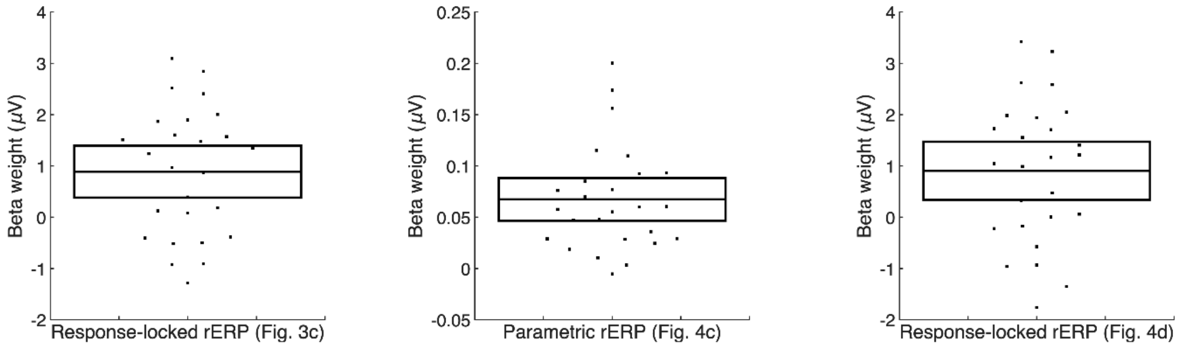

Supplementary Figure 5. Participant rERP scores from the main manuscript. Error bars represent 95% confidence intervals.
